# Molecular Typing and Antimicrobial Susceptibility Profile of *Staphylococcus aureus* Isolates Recovered from Bovine Mastitis and Nasal Samples

**DOI:** 10.3390/ani10112143

**Published:** 2020-11-18

**Authors:** Renata P. Santos, Fernando N. Souza, Ana Claudia D. Oliveira, Antônio F. de Souza Filho, Juliana Aizawa, Luisa Z. Moreno, Adriano F. da Cunha, Adriana Cortez, Alice M.M.P. Della Libera, Marcos B. Heinemann, Mônica M.O.P. Cerqueira

**Affiliations:** 1 Departamento de Inspeção e Produtos de Origem Animal, Escola de Veterinária, Universidade Federal de Minas Gerais, Av. Antônio Carlos 6627, Belo Horizonte 31270-901, Brazil; renatadepaolisantos@gmail.com (R.P.S.); oliveiraacd@gmail.com (A.C.D.O.); adrianofcunha@hotmail.com.br (A.F.d.C.); monicapinhocerqueira@gmail.com (M.M.O.P.C.); 2 Departamento de Clínica Médica, Faculdade de Medicina e Veterinária e Zootecnia, Universidade de São Paulo, Av. Prof. Dr. Orlando Marques de Paiva, 87, Cidade Universitária, São Paulo 05508-270, Brazil; dellalibera@usp.br; 3 Programa de Pós-Graduação em Ciência Animal, Universidade Federal da Paraíba, Areia 58397-000, Brazil; 4 Departamento de Medicina Veterinária Preventiva e Saúde Animal, Faculdade de Medicina e Veterinária e Zootecnia, Universidade de São Paulo, Av. Prof. Dr. Orlando Marques de Paiva, 87, Cidade Universitária, São Paulo 05508-270, Brazil; antoniosf@usp.br (A.F.d.S.F.); juaizawa@gmail.com (J.A.); luisa.moreno@usp.br (L.Z.M.); marcosbryan@usp.br (M.B.H.); 5 Curso de Medicina Veterinária, Universidade Santo Amaro, Rua Prof. Enéas de Siqueira Neto 340, São Paulo 04829-300, Brazil; acortez@prof.unisa.br

**Keywords:** intramammary infection, spa typing, staphylococci, antimicrobial susceptibility, dairy cow

## Abstract

**Simple Summary:**

*Staphylococcus aureus* is a major, prevalent mastitis pathogen, representing a real issue for bovine udder health, with unquestionable importance in human and veterinary medicine. The present study thus aimed to determine the antimicrobial resistance and the diversity of *S. aureus* recovered from transient and persistent intramammary infections and from extramammary niches (e.g., nares/muzzles) in dairy cows. We found that a large proportion of *S. aureus* strains exhibited multidrug resistance to antimicrobials, including resistance to antimicrobials that are critically important to human health. *S. aureus* isolates from transient and persistent IMIs did not differ, suggesting that the persistence of bovine intramammary infections (IMIs) was mainly determined by host factors, although *S. aureus* isolated from extramammary niches are not an important source of *S. aureus* intramammary infections. Furthermore, a discrepancy in antimicrobial resistance between *S. aureus* strains isolated from nares/muzzles and intramammary infections was observed.

**Abstract:**

In the present study, we aimed to determine the antimicrobial resistance and molecular typing of *Staphylococcus aureus* recovered from transient and persistent intramammary infections and nares/muzzles in dairy cows. We investigated the antimicrobial resistance of 189 *S. aureus* strains using a broad antimicrobial susceptibility profile. Furthermore, 107 *S. aureus* isolates were strain-typed using staphylococcal protein-A (spa) typing. A large proportion of strains exhibited multidrug resistance to antimicrobials, including resistance to critically important antimicrobials, although no methicillin-resistant *S. aureus* strains were found. Our study did not strengthen the idea that extramammary niches (i.e., nares/muzzles) are an important source of *S. aureus* for bovine mastitis. A discrepancy in the antimicrobial resistance between *S. aureus* strains isolated from nares/muzzles and milk samples was observed. Furthermore, *S. aureus* isolates from transient and persistent intramammary infections (IMIs) did not differ by spa typing, suggesting that the persistence of bovine IMIs was determined by cow factors. Thus, the high level of multidrug-resistant *S. aureus* found in the two herds, considered together with the predominance of a well udder-adapted *S. aureus* strain, may contribute to our knowledge of the history of the high prevalence of mastitis caused by *S. aureus*, which is of great concern for animal and public health.

## 1. Introduction

Bovine mastitis is the most prevalent and costly disease that affects dairy farming. Moreover, it has great implications for milk production, the quality of milk and dairy products, antimicrobial usage, animal welfare, the environment, and the image of the dairy sector. Among other mastitis pathogens, *Staphylococcus aureus* is a major, prevalent mastitis pathogen, which represents a significant issue for bovine udder health, with unquestionable importance in human and veterinary medicine [1]. Although antibiotic treatment is widely used to fight bovine mastitis, *S. aureus* resistance to antimicrobials not only complicates antimicrobial treatment, but also represents a huge challenge for public health and food security, as cows are the major reservoir for the emergence of *S. aureus* human epidemic clones [2].

Furthermore, from the epidemiological point of view, it is crucial to increase our understanding on the circulating bovine-associated *S. aureus* strains among the cow population. A variety of molecular methods have been extensively used for typing *S. aureus* isolates, of which staphylococcal protein-A (spa) typing is one among the most common. spa typing is suitable for epidemiological studies and produces reproducible, unambiguous, and easily interpreted results [3]. Currently, the growing spa typing database developed by Harmsen et al. [4] is the largest database for typing *S. aureus*, surpassing that of multilocus sequence typing.

Thus, the objective of the present study was to determine the antimicrobial resistance and molecular typing of *S. aureus* strains recovered from transient and persistent intramammary infections and nares/muzzles.

## 2. Materials and Methods 

This study was approved by the Animal Research Ethics Committee of the Federal University of Minas Gerais, Minas Gerais, Brazil, under the protocol number 201/2011. All bacterial strains were collected from two dairy herds with approximately 125 lactating dairy cows per herd between January 2013 and January 2014. Both herds had a high bulk tank milk somatic cell count (≥5.0 × 10^5^ cells mL^−1^) and a history of a high level of mastitis caused by *S. aureus* (as determined by a veterinarian and herd statistics). At the beginning of the study, 24.80% and 15.75% of lactating dairy cows had an intramammary infection (IMI) by *S. aureus* in herds A and B, respectively. The dairy farms located in Minas Gerais state, Brazil, are geographically distant (approximately 450 km apart).

### 2.1. S. aureus Isolates from Milk Samples

We used 182 *S. aureus* isolates previously identified by biochemical tests [1,5]. All *S. aureus* isolates were further identified by matrix-assisted laser desorption ionization time of flight mass spectrometry (MALDI-TOF MS; Bruker Daltonics, Bremen, Germany), as previously described by Nonnemann et al. [6]. Furthermore, *S. aureus* identification was confirmed by polymerase chain reaction (PCR) analysis targeting a portion of the thermonuclease gene conserved in *S. aureus* (nuc) [7]. All *S. aureus* isolates from milk samples were analyzed by an antimicrobial susceptibility test.

The *S. aureus* strains isolated from milk samples were further categorized into those derived from persistent and transient IMIs. A quarter was defined as having an IMI caused by *S. aureus* if ≥100 colony-forming unit colonies mL^−1^ were detected in the milk. An IMI was regarded as transient if *S. aureus* was isolated at only one sampling of the consecutive samplings, in the period between the first and the last samplings. A persistent IMI was assumed if a quarter had an IMI at ≥3 consecutive samplings (at least a one-month interval) caused by the same *S. aureus* strain. From those isolates, we selected 100 *S. aureus* isolates from milk samples according to their epidemiological behavior (transient vs. persistent IMIs) for spa typing.

### 2.2. S. aureus Isolates from Muzzle/Nare Samples

From the same herds and period, the nares/muzzles of dairy cows were sampled by swabbing the muzzle and the inner nares with a single moistened sterile cotton swab, as previously described [8]. Then, the swabs were spread onto the surface of Baird-Parker agar (Oxoid) plates with 5% egg yolk tellurite emulsion and incubated at 37 °C for 24–48 h under aerobic conditions. From each plate, if present, three gray-to-black colonies surrounded by clear zones, as a result of proteolysis and lipolysis, and three gray-to-black colonies without clear zones were selected and transferred separately to microtubes containing 900 μL of brain heart infusion (BHI), and incubated overnight at 37 °C, then glycerol (10% final concentration) was added, and the samples were stored at 80 °C until identification. Afterwards, for bacterial identification, the bacterial isolates (*n* = 159) were spread onto BHI agar plates for 24–48 h at 37 °C. The bacterial isolates were first subjected to Gram staining and catalase and coagulase tests and further identified by MALDI-TOF MS [6]. Furthermore, *S. aureus* identification was confirmed by PCR analysis targeting a portion of the *S. aureus* nuc gene [7]. Among the 159 bacterial isolates, all *S. aureus* isolates identified by both MALDI-TOF MS and PCR as *S. aureus* were subjected to spa typing and antimicrobial susceptibility tests.

### 2.3. Spa Typing

First, deoxyribonucleic acid (DNA) extracted from the bacterial culture in BHI broth by means of a method adapted from the boiling methodology described by Fan et al. [9], where phosphate-buffered saline (PBS PO_4_ 0.01 M, NaCl 0.15 M, pH 7.2) was replaced with tris-Ethylenediaminetetraacetic acid (EDTA) buffer (TE tris-HCl 10 mM, EDTA 1 mM, pH 8.0). The repeated region of *S. aureus* protein A was amplified with the primers previously described by Harmsen et al. [4], and the following PCR conditions were used: heating at 95 ℃ for 5 min, 35 cycles of amplification at 95 ℃ for 45 s, annealing at 60 ℃ for 45 s, extension at 72 ℃ for 90 s, and a final extension at 72 ℃ for 10 min. The DNA sequences were obtained with an ABI-3500 automatic sequencer (Applied Bisystems^®^, Foster, CA, USA). Spa types were determined with the protocol recommended by the Ridom spa Server (http://www.spaserver.ridom.de). The obtained spa-type sequences were analyzed using the spa plugin included in Bionumerics 7.6 (Applied Maths NV, Sint-Martens-Latem, Belgium).

### 2.4. Antimicrobial Susceptibility Tests

A broad antimicrobial susceptibility profile was analyzed by the automated Vitek 2^®^ compact system (BioMériux, Inc., Durham, NC, USA) by determining minimum inhibitory concentration (MIC) using veterinary susceptibility AST-GP69 card (BioMériux, Inc., Durham, NC, USA) panels for Gram-positive bacteria. The following antimicrobials were tested by the Vitek 2^®^ kit: ampicillin/sulbactam, benzylpenicillin, cefoxitin screen, chloramphenicol, clindamycin, inducible resistance to clindamycin, enrofloxacin, erythromycin, fusidic acid, gentamicin, imipenem, kanamycin, marbofloxacin, mupirocin, nitrofurantoin, oxacillin, rifamycin, tetracycline, trimethoprim/sulfamethoxazole, and vancomycin. *S. aureus* isolates were regarded as multidrug-resistant if they were not susceptible to three or more classes of distinct antimicrobials. Furthermore, *S. aureus* resistance to penicillin was excluded from the definition of multidrug-resistance because of the widespread resistance of *S. aureus* to this antimicrobial agent, as proposed by Magiorakos et al. [10]. All isolates were also tested by the Kirby–Bauer disk diffusion technique using disks for oxacillin (1 µg) and cefoxitin (10 UI/30 µg) for the prediction of methicillin-resistant *S. aureus* (MRSA). All antimicrobial susceptibility criteria were interpreted according to the Clinical and Laboratory Standards Institute [11,12] and the European Committee on Antimicrobial Susceptibility Testing [13].

In addition, all *S. aureus* isolates phenotypically regarded as oxacillin- and/or cefoxitin-resistant by MIC using the Vitek 2^®^ compact system or the Kirby–Bauer disk diffusion technique were further investigated by Etest^®^ (bioMérieux, Basingstoke, UK) and the presence of the methicillin resistance gene (mecA and mecC). PCR analysis for the detection of the mecA and mecC genes was performed as previously described by Mehrotra et al. [14] and Paterson et al. [15], respectively.

## 3. Results

In the present study, the identification of 159 bacteria isolates from nasal/muzzle swabs using MALDI-TOF MS resulted in the identification of *Staphylococcus chromogenes* (*n* = 87, 54.72%), *Staphylococcus haemolyticus* (*n* = 21; 13.21%), *Bacillus pumilus* (*n* = 12; 7.55%), *Staphylococcus hyicus* (*n* = 10; 6.29%), *S. aureus* (*n* = 9; 5.66%), *Staphylococcus xylosus* (*n* = 3; 1.89%), *Corynebacterium efficiens* (*n* = 3; 1.89%), *Enterococcus casseli* (*n* = 2; 1.26%), *Enterococcus faecium* (*n* = 2; 1.26%), *Staphylococcus saprophyticus* (*n* = 1; 0.63%), *Staphylococcus warneri* (*n* = 1; 0.63%), *Staphylococcus nepalensis* (*n* = 1; 0.63%), *Macrococcus caseolyticus* (*n* = 1; 0.63%), *Enterococcus mundtii* (*n* = 1; 0.63%), *Arthrobacter gandavensis* (*n* = 1; 0.63%), *Arthrobacter koreensis* (*n* = 1; 0.63%), *Arthrobacter protophormiae* (*n* = 1; 0.63%), *Bacillus subtilis* (*n* = 1; 0.63%), and *Cellulosimicrobium cellulans* (*n* = 1; 0.63%). All bacteria identified as *S. aureus* using MALDI-TOF MS were confirmed by the presence of the nuc gene using PCR.

**Figure 1:**
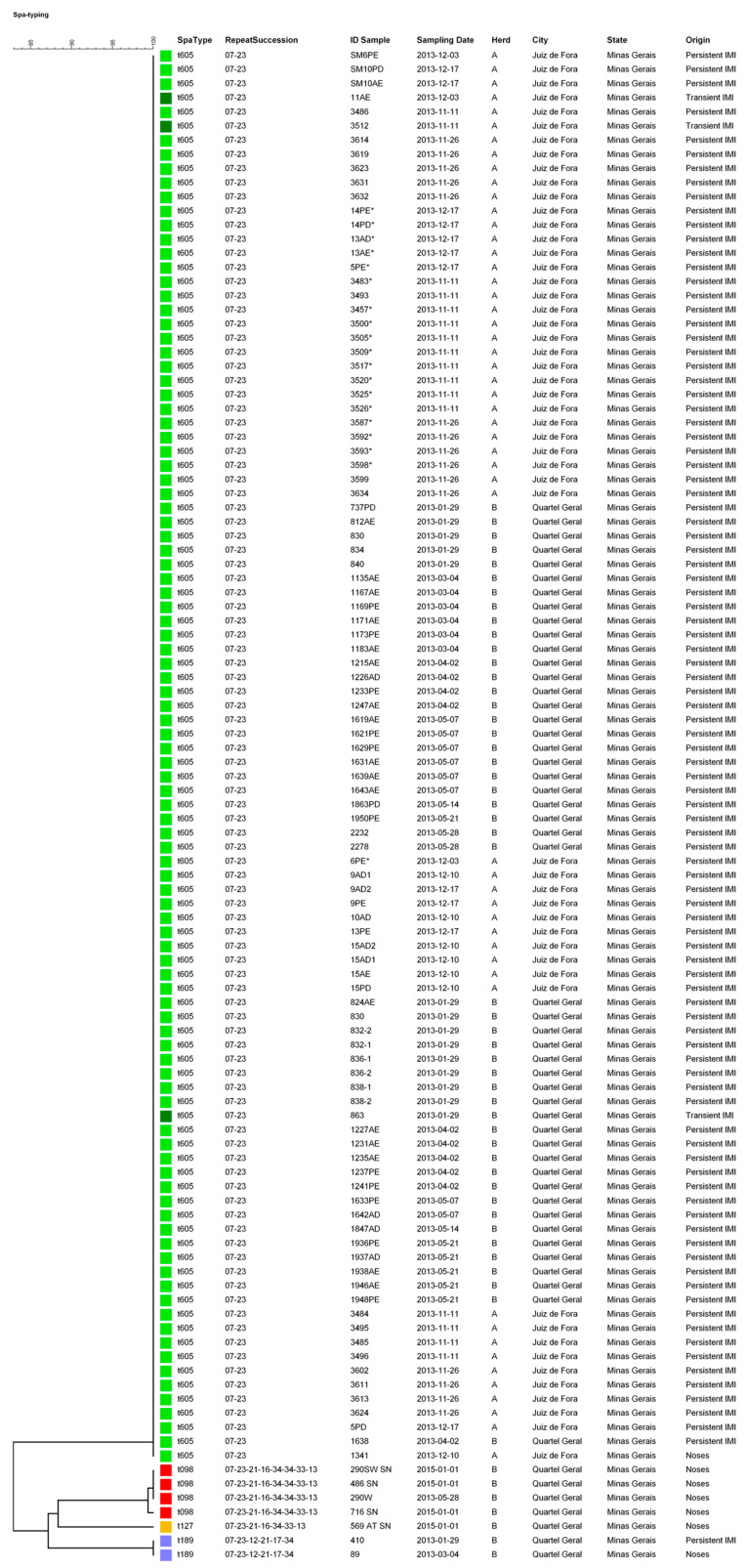
Dendrogram of *Staphylococcus aureus* strains isolated from milk (*n* = 100) and nares/muzzles (*n* = 7), discriminated by spa typing.

Among the 107 *S. aureus* isolates obtained, t605 (93.46%; 99.00% of them from milk), t189 (1.87%; one from milk and one from nares/muzzles), t098 (3.74%; from nares/muzzles) and t127 (0.93%; from nares/muzzles) were molecularly identified (Figure 1).

The antimicrobial susceptibility results by the Vitek 2^®^ Compact system are summarized in Table S1. Our results showed that 46.56% (*n* = 88) of *S. aureus* isolates were not susceptible to at least three distinct classes of antimicrobials. Beyond that, even if we excluded resistance to penicillin, 30 (15.87%) isolates were regarded as multidrug-resistant *S. aureus*. We found eight and four oxacillin-resistant and cefoxitin-resistant *S. aureus* isolates from milk samples using the Vitek 2^®^ Compact system, respectively. Therefore, although most of them (seven and four oxacillin-resistant and cefoxitin-resistant *S. aureus* isolates, respectively) were confirmed by the Kirby–Bauer disk diffusion technique, the resistance to methicillin was not confirmed by the E-test^®^ or the presence of the mecA and mecC genes, suggesting that none of them could be regarded as MRSA. Although a limited number of *S. aureus* strains were isolated from nasal/muzzle samples and characterized in this study, a discrepancy in antimicrobial resistance between *S. aureus* isolated from nares/muzzles and milk samples was observed. For instance, while a great proportion of the *S. aureus* isolates from milk samples were resistant to benzylpenicillin (94.50%), gentamycin (80%), and tetracycline (43.96%), none of the *S. aureus* isolates from nares/muzzles were resistant to these antimicrobials. In contrast, the *S. aureus* isolates from nares/muzzles showed resistance to clindamycin and erythromycin (Table S1).

## 4. Discussion

Apart from the few available studies that have focused on the ecology of *S. aureus* [8,16] and those that have investigated non-*aureus* staphylococci isolated from body sites [17], information on the frequency of bacterial isolates from nares/muzzles in dairy cows is limited. Although *S. aureus* colonization of nares represents a primary reservoir of this pathogen in humans, nares/muzzles did not appear to be a major reservoir for *S. aureus* in dairy cows, corroborating the findings of Capurro et al. [16]. Rather, nares/muzzles appeared to be mainly colonized by non-*aureus* staphylococci in dairy cows.

Furthermore, our study did not strengthen the idea that extramammary niches (i.e., nares/muzzles) are an important source of *S. aureus* strains that cause persistent IMIs in dairy cows. In agreement with our results, Leuenberger et al. [18] reported that the *S. aureus* genotype B is highly associated with the mammary gland, whereas other *S. aureus* genotypes (e.g., C and R) that cause sporadic and noncontagious IMI were the most abundant in extramammary niches, such as the hocks, teat skin, nostrils, and perineum. The only *S. aureus* strain (ST89) originating from the nose was also detected in milk samples, and the latter was associated with a transient IMI in another cow, indicating that if it does cause an IMI, it is likely associated with a sporadic and noncontagious IMI. Moreover, one *S. aureus* strain isolated from herd A was the same strain that caused persistent IMI, suggesting that this strain might adapt to extramammary niches and colonize them, as previously suggested for teat skin [19]. Thus, even though *S. aureus* can colonize extramammary niches of dairy cows (i.e., nares/muzzles) [8,16], our study showed that the udder is the most important reservoir of these bacteria, which also substantiates their contagious behavior.

We also showed that one *S. aureus* strain (spa type t605) was widespread in the two herds investigated, although the herds are geographically distant. This *S. aureus* strain was responsible for almost all IMIs, probably because it is well adapted to the udder, leading to the persistence of IMIs. Thus, our results are in agreement with other reports that indicated that a few udder-adapted *S. aureus* types with broad geographic distribution are responsible for most of the IMIs in dairy herds [20].

Another important finding was that the herd that only had one *S. aureus* strain was a closed herd, containing no cows that had been purchased from other herds in recent decades, in contrast to the other herd, an open herd, in which new *S. aureus* strains could be introduced by animals from foreign herds. We identified an additional *S. aureus* strain in the open herd. Although the owner of the open herd acquired several dairy cows from distinct dairy herds (personal communication), the most udder-adapted *S. aureus* strain might spread more efficiently, preventing the spread of other less well-udder adapted (i.e., opportunistic) *S. aureus* strains. In the present study, the *S. aureus* ST605 clone was the most important clone related to bovine IMIs, which is in agreement with another Brazilian study [21]. To the best of our knowledge, this study is the first report of the isolation of the *S. aureus* ST098 clone from bovine samples. In this study, the *S. aureus* ST098 clone was the most common *S. aureus* strain isolated from nasal samples. We also demonstrated that the persistence of bovine IMIs was probably mainly determined by host factors, most likely related to innate and adaptative immune responses, as the same *S. aureus* strain (ST605 clone) caused persistent and transient IMIs.

The antimicrobial susceptibility results from the Vitek 2^®^ Compact system are summarized in Table S1. Our results showed that 46.56% (*n* = 88) of *S. aureus* isolates were not susceptible to at least three distinct classes of antimicrobials. Beyond that, even if we excluded resistance to penicillin, as proposed by Magiorakos et al. [10], 30 (15.87%) isolates were regarded as multidrug-resistant *S. aureus*. A high proportion of *S. aureus* isolates originated from IMIs harboring resistance to tetracycline and penicillin has been previously observed in Brazil [22,23], which corroborates our findings. Nevertheless, our study showed a high level of resistance to gentamycin in *S. aureus* originating from IMIs, although gentamycin resistance frequencies reported are generally low [22,23]. The high level of multidrug resistant *S. aureus* found here, together with the predominance of an udder-adapted strain, may contribute to our knowledge of the history of the high level of mastitis caused by this pathogen, as the existence of penicillin-resistant *S. aureus* is related to low cure rates for *S. aureus* IMI treatment with either β-lactam or non-β-lactam antimicrobials [24]. Although no MRSA was detected here, our study showed that the *S. aureus* isolates from IMIs had considerable resistance to antimicrobials, including resistance to critically important antimicrobials, such as macrolides (e.g., erythromycin) and glycopeptides (e.g., vancomycin). Moreover, some intermediate resistance to critically important antimicrobials such as the quinolone group (e.g., marbofloxacin and enrofloxacin) was also found. Altogether, our data emphasize that *S. aureus* IMIs are concerning to animal and public health. 

We hypothesize that the divergence in antimicrobial resistance could be attributed to, at least in part, the distinct history of exposure to antimicrobials between *S. aureus* isolated from noses/muzzles compared to those isolated from milk samples. For instance, *S. aureus* isolates from noses/muzzles may have been exposed to antimicrobials (e.g., macrolides) used systemically since the early stages of life (e.g., for respiratory diseases and diarrhea treatment), resulting in exposure for a longer time (since colonization). These antimicrobials are totally different from the antimicrobials which are mainly used locally for mastitis treatment (e.g., β-lactams) after the first parturition.

## 5. Conclusions

Overall, we can conclude that *S. aureus* strains isolated from noses/muzzles displayed distinct strain-typing and antimicrobial resistance profiles from those isolated from IMIs. Nonetheless, *S. aureus* isolated from transient and persistent *S. aureus* IMIs did not reveal divergent strain typing patterns, suggesting that the persistence of *S. aureus* IMIs is mainly determined by host factors.

## Supplementary Materials

Table S1 is available online at https://www.mdpi.com/2076-2615/10/11/2143/s1, Table S1. Antimicrobial susceptibility profile of the *Staphylococcus aureus* isolates from milk and nasal/muzzle swab samples determined using the automated Vitek 2^®^ compact system by minimum inhibitory concentration.

## References

[1] Cunha A., Andrade H., Souza F., Júnior L.F., Rosa D., Sanchez E.R., Gidlund M., Goto H., Brito M., Guimarães A. (2020). Comparison of antibody repertories against Staphylococcus aureus in healthy and infected dairy cows with a distinct mastitis history and vaccinated with a polyvalent mastitis vaccine. J. Dairy Sci..

[2] Richardson E.J., Bacigalupe R., Harrison E.M., Weinert L.A., Lycett S., Vrieling M., Robb K., Hoskisson P.A., Holden M.T., Feil E.J. (2018). Gene exchange drives the ecological success of a multi-host bacterial pathogen. Nat. Ecol. Evol..

[3] Štěpán J., Pantůček R., Doškař J. (2004). Molecular diagnostics of clinically important staphylococci. Folia Microbiol..

[4] Harmsen D., Claus H., Witte W., Rothgänger J., Turnwald D., Vogel U. (2003). Typing of Methicillin-Resistant Staphylococcus aureus in a University Hospital Setting by Using Novel Software for spa Repeat Determination and Database Management. J. Clin. Microbiol..

[5] Souza F.N., Cunha A.F., Rosa D.L., Brito M.A.V., Guimarães A.S., Mendonça L.C., Souza G.N., Lage A.P., Blagitz M.G., Della Libera A.M. (2016). Somatic cell count and mastitis pathogen detection in composite and single or duplicate quarter milk samples. Pesquisa Veterinária Brasileira.

[6] Nonnemann B., Lyhs U., Svennesen L., Kristensen K.A., Klaas I.C., Pedersen K. (2019). Bovine mastitis bacteria resolved by MALDI-TOF mass spectrometry. J. Dairy Sci..

[7] Sasaki T., Tsubakishita S., Tanaka Y., Sakusabe A., Ohtsuka M., Hirotaki S., Kawakami T., Fukata T., Hiramatsu K. (2010). Multiplex-PCR Method for Species Identification of Coagulase-Positive Staphylococci. J. Clin. Microbiol..

[8] Roberson J.R., Fox L.K., Hancock D.D., Gay J.M., Besser T.E. (1994). Ecology of *Staphylcoccus aureus* isolated from various sites on dairy farms. J. Dairy Sci..

[9] Fan H.H., Kleven S.H., Jackwood M.W. (1995). Application of Polymerase Chain Reaction with Arbitrary Primers to Strain Identification of Mycoplasma gallisepticum. Avian Dis..

[10] Magiorakos A.-P., Srinivasan A., Carey R., Carmeli Y., Falagas M., Giske C., Harbarth S., Hindler J., Kahlmeter G., Olsson-Liljequist B. (2012). Multidrug-resistant, extensively drug-resistant and pandrug-resistant bacteria: An international expert proposal for interim standard definitions for acquired resistance. Clin. Microbiol. Infect..

[11] CLSI (2018). Performance Standards for Antimicrobial Disk and Dilution Susceptibility Test for Bacteria Isolated from Animals.

[12] CLSI (2018). Performance Standards for Antimicrobial Disk and Dilution Susceptibility Test for Bacteria Isolated from Animals.

[13] EUCAST (2019). The European Committee on Antimicrobial Susceptibility Testing. Breakpoints Tables for Interpretation of MICs and Zone Diameters–Version 9.0. http://www.eucast.org/fileadmin/src/media/PDFs/EUCAST_files/Breakpoint_tables/v_9.0_Breakpoint_Tables.pdf.

[14] Mehrotra M., Wang G., Johnson W.M. (2000). Multiplex PCR for Detection of Genes forStaphylococcus aureus Enterotoxins, Exfoliative Toxins, Toxic Shock Syndrome Toxin 1, and Methicillin Resistance. J. Clin. Microbiol..

[15] Paterson G.K., Larsen A.R., Robb A., Edwards G.E., Pennycott T.W., Foster G., Mot D., Hermans K., Baert K., Peacock S.J. (2012). The newly described mecA homologue, mecALGA251, is present in methicillin-resistant Staphylococcus aureus isolates from a diverse range of host species. J. Antimicrob. Chemother..

[16] Capurro A., Aspán A., Unnerstad H.E., Waller K.P., Artursson K. (2010). Identification of potential sources of Staphylococcus aureus in herds with mastitis problems. J. Dairy Sci..

[17] Adkins P.R.F., Dufour S., Spain J., Calcutt M., Reilly T., Stewart G., Middleton J. (2018). Molecular characterization of non-aureus Staphylococcus spp. from heifer intramammary infections and body sites. J. Dairy Sci..

[18] Leuenberger A., Sartori C., Boss R., Resch G., Oechslin F., Steiner A., Moreillon P., Graber H. (2019). Genotypes of Staphylococcus aureus: On-farm epidemiology and the consequences for prevention of intramammary infections. J. Dairy Sci..

[19] Paduch J.-H., Krömker V. (2011). Colonization of the teat skin and the teat canal of lactating dairy cattle by mastitis pathogens. Tierärztl. Prax. Ausg. G: Großtiere/Nutztiere.

[20] Srednik M.E., Usongo V., Lépine S., Janvier X., Archambault M., Gentilini E.R. (2018). Characterization of Staphylococcus *aureus* strains isolated from mastitis bovine milk in Argentina. J. Dairy Res..

[21] Silva N., Guimarães F., Manzi M., Budri P., Gómez-Sanz E., Benito D., Langoni H., Rall V., Torres C. (2013). Molecular characterization and clonal diversity of methicillin-susceptible Staphylococcus aureus in milk of cows with mastitis in Brazil. J. Dairy Sci..

[22] Mesquita A.A., Rocha C.M., Bruhn F.R., Custódio D.A., Braz M.S., Pinto S.M., Silva D.B., Da Costa G.M. (2019). Staphylococcus aureus and Streptococcus agalactiae: Prevalence, resistance to antimicrobials, and their relationship with the milk quality of dairy cattle herds in Minas Gerais state, Brazil. J. Pesq. Vet. Bras..

[23] Rabello R.F., Bonelli R.R., Penna B.A., Albuquerque J.P., Souza R.M., Cerqueira A.M.F. (2020). Antimicrobial Resistance in Farm Animals in Brazil: An Update Overview. Animals.

[24] Barkema H.W., Schukken Y., Zadoks R. (2006). Invited Review: The Role of Cow, Pathogen, and Treatment Regimen in the Therapeutic Success of Bovine Staphylococcus aureus Mastitis. J. Dairy Sci..

