# Supplementary material for: Molecular Typing and Antimicrobial Susceptibility Profile of Staphylococcus aureus Isolates Recovered from Bovine Mastitis and Nasal Samples
Source: Animals (Basel). 2020 Nov 18;10(11):2143. doi: 10.3390/ani10112143 (PMC7698789; doi:10.3390/ani10112143)
Supplement: Supplementary file 1 [file animals-10-02143-s001.pdf]

Table S1. Antimicrobial susceptibility profile of the *Staphylococcus aureus* isolates from milk and nasal/muzzles swabs samples performed by the automated Vitek 2® compact system by minimum inhibitory concentration.

Antimicrobial (range of concentrations tested)	MIC BP (μg mL^{-1})	Origin Milk = 182 Swab = 7	Resistant (%)	Number and percentage of isolates at each indicated MIC value (μg mL^{-1})																				
				0.03	0.06	0.25	0.5	1	2	4	5	8	10	16	20	32	40	64	80	128	160	256	320	512
Fusidic acid[2] (0.5 – 32 μg mL^{-1})	≥ 1	Milk	3.85	3.85	–	–	–	173 (95.05)	2 (1.10)		**1 (0.55)**	–	**1 (0.55)**	–	**1 (0.55)**	–	**4 (2.20)**	–	1_	–	–	–	–	–
		Swab	0	0				7 (100)																
Ampicillin/Sullbactam[1] (2.0 – 32 μg mL^{-1})	≥ 16	Milk	1.10	1.10	–	–	–	–	–	175 (96.15)	4 (2.20)	–	1 (0.55)	–	**2 (1.10)**	–		–	–	–	–	–	–	–
		Swab	0	0						7 (100)														
Benzylpenicillin[1] (0.03125 – 0.5 μg mL^{-1})	≥ 0.25	Milk	94.50	94.50	9 (4.95)	1 (0.55)	**1 (0.55)**	**171 (93.95)**	–	–	–	–	–	–	–	–	–	–	–	–	–	–	–	–
		Swab	0	0	7 (100)																			
Clindamycin[1] (0.25 – 8.0 μg mL^{-1})	≥ 4	Milk	2.20	2.20	–	–	175 (96.15)	1 (0.55)		**2 (1.10)**		–	**4 (2.20)**	–	–	–	–	–	–	–	–	–	–	–
		Swab	14.29	14.29			6 (85.71)						**1 (14.29)**											

Inducible resistance to clindamycin[1]		Milk	0	0																				
		Swab	0	0																				
Chloramphenicol[1] (4.0 – 64 µg mL^{-1})	≥ 32	Milk	0	0	–	–	–	–	–		102 (56.04)	–	79 (43.41)	1 (0.55)		–		–		–	–	–	–	–
		Swab	0	0							1 (14.29)		6 (85.71)											
Enrofloxacin[1] (0.5 – 4.0 µg mL^{-1})	≥ 4	Milk	0	0	–	–	–	172 (94.50)	**8 (4.40)**	**2 (1.10)**		–	–	–	–	–	–	–	–	–	–	–	–	–
		Swab	0	0				7 (100)																
Erythromycin[1] (0.25 – 8.0 µg mL^{-1})	≥ 8	Milk	3.85	3.85	–	–	174 (95.60)	1 (0.55)				–	**7 (3.85)**	–	–	–	–	–	–	–	–	–	–	–
		Swab	14.29	14.29			6 (85.71)						**1 (14.29)**											
Gentamicin[1] (0.5 – 16 µg mL^{-1})	≥ 16	Milk	80	80	–	–		101 (55.49)	1 (0.55)						**80 (43.96)**									
		Swab	0	0				7 (100)																

Imipenem<br>(1.0 – 16 µg mL^{-1})	NA	Milk<br>Swab			–	–	–	–	182 (100)<br>7 (100)			–		–		–	–	–	–	–	–	–	–	–
Kanamycin[1]<br>(4.0 – 64 µg mL^{-1})	≥ 64	Milk<br>Swab	43.96<br>0	43.96<br>0	–	–	–	–	–	–	101 (55.49)<br>7 (100)	–	1 (0.55)	–		–		–	**80 (43.96)**	–	–	–	–	–
Marbofloxacin[1]<br>(0.5 – 4.0 µg mL^{-1})	≥ 4	Milk<br>Swab	0<br>0	0<br>0	–	–		173 (95.05)<br>6 (85.71)	**3 (1.65)**<br>**1 (14.29)**	**6 (3.30)**		–	–	–	–	–	–	–	–	–	–	–	–	–
Mupirocin[2]<br>(2.0 – 512 µg mL^{-1})	≥ 256	Milk<br>Swab	1.10<br>0	1.10<br>0	–	–	–	–	–	172 (94.50)<br>7 (100)		–		–		–		–	**8 (4.40)**	–		–		–
Nitrofurantoin[1]<br>(16.0 – 512 µg mL^{-1})	≥ 128	Milk<br>Swab	0.55<br>14.29	0.55<br>14.29	–	–	–	–	–	–	–	–	–	–	174 (95.60)<br>6 (85.71)		4 (2.20)	–		–	**3 (1.65)**	–	**1 (0.55)**<br>**1 (14.29)**	–
Rifamycin[1]<br>(0.5 – 32 µg mL^{-1})	≥ 4	Milk<br>Swab	2.2<br>0	2.2<br>0	–	–	–	175 (96.15)<br>7 (100)	2 (1.10)	**1 (0.55)**		–		–	**1 (0.55)**	–	**3 (1.65)**	–	–	–	–	–	–	–

Tetracycline[1] (1.0 – 16 µg mL^{-1})	≥ 16	Milk<br>Swab	43.96<br>0	43.96<br>0	–	–	–	–	94 (51.64)<br>6 (85.71)	6 (3.30)<br>1 (14.29)	1 (0.55)	–	**1 (0.55)**	–	**80 (43.96)**	–	–	–	–	–	–	–	–	–
Trimethroprim/ sulfamethaxazole[2] (10.0 – 320 µg mL^{-1})	≥ 80	Milk<br>Swab	0.55<br>0	0.55<br>0	–	–	–	–	–	–	–	–	–	178 97.80)<br>1 (85.71)	–	3 (1.65)<br>1 (14.29)	–		–		–		–	**1 (0.55)**
Vancomycin[1] (0.5 – 32 µg mL^{-1})	≥ 16	Milk<br>Swab	3.30<br>0	3.30<br>0	–	–	–	88 (48.35)<br>5 (71.43)	78 (42.86)<br>2 (28.57)	10 (5.49)		–		–		–	**6 (3.30)**	–	–	–	–	–	–	–
Oxacillin[1] (0.25 – 4.0 µg mL^{-1})	≥ 4	Milk<br>Swab	4.40<br>0	4.40<br>0	–	–	162 (89.01)<br>7 (100)	11 (6.04)		1 (0.55)	**8 (4.40)**	–	–	–	–	–	–	–	–	–	–	–	–	–
Cefoxitin screening	≥ 8	Milk<br>Swab	2.20<br>14.29	2.20<br>14.29																				

MIC: minimal inhibitory concentration. BP: MIC breakpoint, which is defined as the lowest concentration of an antimicrobial necessary to inhibit visible growth. [1]MIC breakpoints established by the Clinical & Laboratory Standards Institute (CLSI) guidelines (CLSI, 2018a and 2018b). [2]MIC breakpoints established by the European Committee on Antimicrobial Susceptibility Testing (EUCAST, 2019). Values in bold indicate intermediate resistance to that antimicrobial. Values in bold and red indicate resistance to that antimicrobial. Swab: nasal swab. NA: not available. Dashes indicate values not tested for the indicated antimicrobial.
